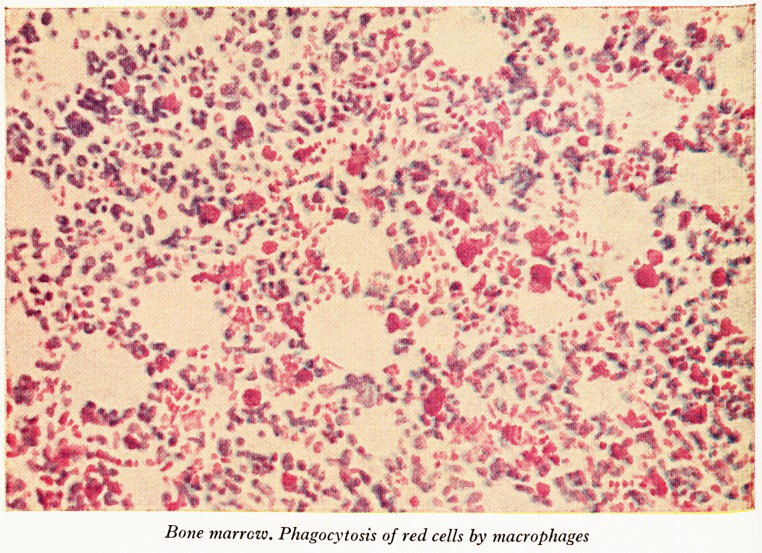# Paroxysmal Nocturnal Haemoglobinuria

**Published:** 1963-07

**Authors:** T. F. Hewer


### PAROXYSMAL NOCTURNAL HAEMOGLOBINURIA

*A Clinico-Pathological Conference held in the University of Bristol on the 6th November, 1962*

CHAIRMAN: PROFESSOR T. F. HEWER

*Dr. Crow:* This lady was 57 at the time of death and although her early history is a little scanty it seems likely that the disease which ultimately caused her death first presented around the age of 21. We have nothing like complete notes up to 1953, but even so her notes weigh 3 lb 10 oz!

She had an unfortunate social background. She had a hard childhood, apparently suffered from jaundice at the age of 10 and is said to have been "yellow" throughout her adolescence, though what that means I do not know. In 1926 at the age of 21 she was seen at the General Hospital and was treated with liver injections, a diagnosis having apparently been made of pernicious anaemia. In 1932 the notes say she had an episode of passing black water, but we have no other record of that. In 1934 during her first pregnancy she is said to have had haematuria for six months and this was sufficiently severe to require transfusion. In 1940 during her second pregnancy she again had persistent prolonged haematuria, which again required transfusion. In 1941 she was seen in Outpatients and apparently the diagnosis of pernicious anaemia was still being held. In 1945, at the age of 40, she had further investigations done, about which Dr. Tovey will tell you presently, and a therapeutic abortion was advised. She was investigated again in 1948 when she was again found to be anaemic. On this occasion it was definitely established that this patient had a haemolytic anaemia. There was an excess of urobilin in the urine. Her reticulocytes were persistently raised and she had both haemoglobin and methaemalbumin in the blood serum. At that time her Hb was 50 per cent and the total white cell count was 2,300, 50 per cent of which were polymorphs.

There appears to be a gap in the notes, for although she did attend the Bristol Royal Infirmary in 1949 I have no record of the findings. In 1953 she was re-admitted for investigation and was found to have an iron deficiency anaemia. On this occasion there was no haemolysis going on. Fractional test meal revealed that she had an achlorhydria but this did not respond to histamine injection. A few months later she was re-admitted during an episode of haemolytic anaemia; she was pale, Hb 50 per cent, she had raised bilirubin in the blood, her urine contained urobilin, haemoglobin and haemosiderin granules, there was some albuminuria as well and there were some granular casts. Her blood was investigated to re-establish the nature of this haemolysis. The Donath-Landsteiner test for paroxysmal cold haemoglobinuria was negative; tests for various antibodies, direct and indirect antiglobulins, warm and cold agglutinins, warm and cold haemolysins were all negative. The red cell fragility was normal. Ham's test—a specific test for paroxysmal nocturnal haemoglobinuria—was positive, and Schumm's test established the fact that there was methaemalbumin present in the blood serum. The blood W.R. and Kahn were negative. It was therefore established that she had paroxysmal nocturnal haemoglobinuria.

From then on she had increasingly frequent admissions to hospital on account of anaemia due to recurrent bouts of haemolysis. During these attacks, which usually began at night, she would get up in the morning and pass very dark urine. During a bad bout the haemoglobinuria was persistent throughout the day, but it was usually worse at night. I have a slide which shows her urine during these haemolytic phases (Plate XVIII): in the morning the urine looks like treacle, by 2 p.m. it looks like urine. The following day it looked dark brown in the morning—there had not been such severe haemolysis—and cleared again during the day. This is a good illustration of how the haemolysis occurs and why it is called paroxysmal nocturnal haemoglobinuria.

On many occasions the haematologists have recorded from blood films that the platelets were scanty, on several occasions they were counted and were found to be as low as 20,000/c.mm; on other occasions they were 70,000/c.mm but the very low counts were characteristic. On many occasions she had a neutropenia as well, the total white count being in the range of 2–4,000 and the polymorph count usually about 50 per cent of this. The notes show that on several occasions no precipitating factors were known, but it was recognised that many of her episodes of haemoglobinuria were precipitated by some specific event. Infection seems to have been one of the major precipitants. In 1954 a trichomonas vaginitis developed just before a haemolytic episode.

In 1960 she had an acute urinary infection and after a week developed severe haemoglobinuria, the Hb dropping down to about 30 per cent. Further episodes of urinary infection occurred, sometimes coliform which was treated successfully, but on the next admission a catheter specimen of urine was sterile. She had bouts of boils. On one occasion a dental extraction, followed very soon by an incision for an abscess of the foot, precipitated an attack. On another occasion, very interestingly, an episode began immediately after she had had an accident in the street. She was not injured but received a severe shock. She herself is recorded as saying worry would bring on an attack. This poor woman had a very unfortunate background throughout her life, her marriage was unhappy and she had a hard time, so that she had plenty of worry which might well have precipitated attacks.

Another feature is the frequency with which she got thrombophlebitis at the site of transfusion. The usual treatment when she came in to hospital suffering from haemolysis was complete rest and blood transfusion, which was given in the form of packed washed red cells, the reason for which I hope Dr. Raper will shortly tell us. She was given repeated courses of iron, sometimes intravenously, sometimes intramuscularly and for long continuous periods by mouth. The reason for her fairly constant iron deficiency was that she was losing a lot of iron in her urine. She had fairly frequent episodes of phlebitis at the site of transfusion, rather more frequently than can be explained by careless technique. In fact she illustrates during this long course the apparently characteristic feature of this disease:—a liability to infections of all kinds, to phlebitis, not necessarily induced by transfusion but spontaneous episodes of thrombophlebitis, and of course there were her attacks precipitated by pregnancy.

In August 1961 she was admitted following a fairly prolonged bout of haemoglobinuria, without any apparent precipitating cause. She was transfused, but remained pretty unwell. She was treated for the first time with Diamox. Previously she had frequently and for continuous periods been treated with alkalis, the object of which was to maintain the blood pH on the alkaline side. On this occasion Diamox was tried, as another means of achieving the same end. Three days after starting the Diamox she developed a left hemiplegia which was attributed to cerebral thrombosis. From this she made a very slow but eventually fair recovery and she was able to get about.

Seven months later, in March 1962 she was re-admitted for assessment under

Dr. Cates. She was not particularly anaemic, Hb was 67 per cent, total neutrophil polymorphs 3,000, platelets were scanty on the film. The urine was sterile. In April she was re-admitted as an emergency. She had developed diarrhoea with incontinence of faeces for seven days and then severe haemoglobinuria. On admission she was very ill, severely anaemic, slightly icteric. On this occasion, I think for the first time, she had widespread purpura, her blood pressure was only 100 mm. systolic, 60 diastolic, she was clinically mildly shocked, she had moderate tachycardia, Hb was 29 per cent. She was treated with blood transfusion, which brought her Hb to 56 per cent. Her platelets were noted to be scanty. She had a severe coliform infection of the urine. She remained very ill and developed overt rectal bleeding, or gastro-intestinal bleeding anyway and in spite of further transfusions she remained anaemic; she became drowsy and finally comatose. Her urine function appeared to be inadequate. Her blood urea rose to 234 mgm per cent some five to six days after admission. She was hypotensive, which was hardly attributable to actual loss of blood volume. At this time she was thought clinically to be suffering from what is called an aplastic crisis. Her bone marrow, which for all these years had been struggling in the face of severe continuous loss of red cells, packed up and would not work any more. Her white cells were low, her platelets were low. In an effort to improve her malfunction she was given steroids in quite heavy doses and was also given folic acid but it made no difference. She died in coma on the 30th April 1962, seven days after her final admission.

And now I think possibly Dr. Tovey wants to tell us of his early knowledge of this patient, before any of the BRI notes were made, which I have just detailed.

*Dr. Tovey:* As Dr. Crow has said I saw this patient in 1945. I shall never forget the first time I saw her because I think she is the only patient I have ever seen wearing cricket flannels—not the patient, I was wearing the flannels! I received a message towards the end of a cricket match asking if I would see a patient in the General Hospital who had been given an incompatible transfusion. The patient had been transfused with a bottle of packed red cells because of an anaemia following an abortion and was now passing haemoglobin in her urine. When I saw the patient I confirmed that she had haemoglobinuria but was otherwise very well. This suggested that she had probably not had an incompatible transfusion, and a few simple tests quickly excluded incompatibility. The patient was group B but, because the Army required all blood of this group for plasma, she had been transfused with group O blood. It was possible therefore to check whether the reaction was due to an incompatible transfusion by grouping a sample of the patient's blood taken after the transfusion and examining it for group O cells. These tests showed many group O transfused cells to be present, thus excluding an incompatible transfusion and making one suspect that the patient had an underlying haemolytic anaemia. As you know, the plasma of group O blood contains anti-A and anti-B agglutinins, and although the patient had been given packed cells there were sufficient anti-B agglutinins present in the transfused plasma to react with the recipient's own group B cells and cause some of them to haemolyse. We did not know it at that time, but this is the basis of a useful diagnostic test for paroxysmal nocturnal haemoglobinuria. The cells of a normal individual who is group B are agglutinated by an anti-B grouping serum, but if the patient is suffering from paroxysmal nocturnal haemoglobinuria, the grouping tests show not only agglutination but also haemolysis, although there is no complement in the system.

I next saw the patient in 1948 when she was having a further transfusion, and see from my notes that at that time she had a reticulocytosis of about 10 per cent. The transfusion was again complicated by haemoglobinuria and we considered that she she was probably suffering from paroxysmal nocturnal haemoglobinuria, although the laboratory tests at that time did not establish any autohaemolysis. After this, as

PLATE XVIII

*Successive urine specimens; nocturnal haemoglobinuria*

PLATE XIX

*Kidney. Brown cortex, with pyelonephritic abscess*

PLATE XX

*Renal cortex. Pronounced Prussian blue reaction in convoluted tubules*

PLATE XXI

*Bone marrow. Phagocytosis of red cells by macrophages*

Dr. Crow has mentioned, she went on to have many transfusions. It was found that these were generally accompanied by reactions but that the reactions could be avoided by transfusing *washed* packed cells. It used to be thought that the washing removed some protein factor to which such patients had become sensitised. We now know, however, that patients receiving repeated transfusions may develop leucocyte as well as red cell antibodies, and will react with rigors and unpleasant temperature reactions unless you give them washed leucocyte-free blood.

*Dr. Raper:* You have heard from the history how many strange incidents occurred, and I should like first to refer to the onset, which was very gradual, and it was a long time before the diagnosis was made. This is quite common in paroxysmal nocturnal haemoglobinuria because such things as Dr. Crow mentioned, depression of leucocytes and depression of platelets, may occur quite early, and the patient may be suspected of having an aplastic anaemia. I should like also to mention the question of iron deficiency; you will remember that in an ordinary haemolytic anaemia you would not expect iron deficiency to develop because the blood is destroyed within the body; the products are retained and are used again. How is it that this does not happen in this condition? The answer is that in a disease like congenital spherocytosis the destruction of red cells occurs within the reticuloendothelial system, in the spleen for example, and there the haemoglobin is immediately converted and the breakdown products are used again; but in paroxysmal nocturnal haemoglobinuria the red cells are broken down in the blood and the blood stream is flooded with haemoglobin, the amount being such that it escapes through the kidneys as visible haemoglobin, which we see in the urine; and some of it is also retained in the convoluted tubules (Plate XX). This stuff in the convoluted tubules which contains much iron is dropped out cell by cell long after the bout of haemoglobinuria is past. This of course is the basis of one of the tests for a chronic recurrent haemoglobinuric disease. If you look at a deposit of the urine which has been stained with Prussian blue you will find there little granules of iron in cells and outside cells. It is the loss of these hour after hour that drains the person of iron, as well as the actual loss of haemoglobin. So that explains why this differs from other haemolytic anaemias in the development of iron deficiency, and there were times when a confident diagnosis of iron deficiency was made for this lady. Now you will be asking "What is the defect in this sort of patient?" As we have been told, her red cells are lysed in the blood; her own serum will cause this haemolysis and so will that of an ordinary person. The haemolysis will not take place after the serum has been heated and so some labile factor must be important in this reaction. The reaction is more sensitive if the serum is made a little acid, and this is done with N/50 hydrochloric acid. That is the basis of what is known as Ham's test. Ham actually did it with lactic acid, not with hydrochloric. The test is quite simple. We take the serum of the patient or normal serum and acidify it, and then add some of the patient's cells that have been washed, and use some normal cells as a control, incubate the tubes and look for haemolysis. If we heat the serum before doing the test we do not get haemolysis; this would distinguish paroxysmal nocturnal haemoglobinuria from haemolysis that occurred from some other cause, such as spherocytosis. It is clear that because normal people's serum will lyse the cells of these people we have to look for the defect in the cells and not in the serum. People have looked for the defect and no-one knows exactly what it is. It is fairly clear that it lies in the stroma of the red cells and it is thought to be there because the lipo-protein of the red cells is disturbed in paroxysmal nocturnal haemoglobinuria.

It has also been shown recently that there is a disturbance in the enzymes of the red cells, strikingly enough in the cholinesterase; so probably this defect, the reduction of the enzyme, has something to do with the disturbance of the red cells. Another striking fact is that the red cells of all ages, young and old alike, have this defect, whereas normally you would expect it only in the old red cells. These defects are known but we cannot add them up to very much.

This lady had been receiving washed cells since 1956 and had not had any serious reaction in that time. People have tried to treat this condition with anticoagulants because it was noticed that when clotting occurred in the body lysis was increased; and this lady had some cerebral accident involving coagulation which may have determined one of her attacks. People also found that the addition of thrombin to the reacting mixture increased the lysis but this has been shown not to be due to the thrombin. So in fact the use of anticoagulants, although started enthusiastically as usual, was later shown to be of no use. Diamox which was mentioned by Dr. Crow has also been shown to be useless. This is not surprising because someone in America in 1953 put one of these patients into a Drinker respirator throughout the night, adjusting the respirator so that the daily respiratory movement was imitated, and despite this the patient got haemoglobinuria during the night. So although the patient could not retain any more $CO_2$ than during the day, haemolysis still occurred.

*Dr. Lloyd:* First I will tell you what I found, and then I will try and explain what it means. She had severe purpura all over the body, due to a deficiency of platelets. There was a distinct diminution in the number of megakaryocytes in her bone marrow. Those present appeared to be rather primitive, so I do not think she was forming platelets in the normal fashion. In the serious cavities there were petechial haemorrhages, particularly in the pericardial cavity, and in the meninges she had severe subdural and slight subarachnoid haemorrhage. The heart, blood vessels, larynx, trachea, bronchi and lungs were all normal. The spleen weighed 165 g, which is only a little bit more than normal. It was soft and rather purple in colour but the Prussian blue test showed that it contained iron. The lymph nodes were normal. The bone marrow was deep brownish-red throughout the whole of the femur. It was soft and there was no sclerosis of the bone itself. The liver was not enlarged. There was no evidence of centrilobular necrosis (I will tell you about that presently). The Prussian blue test showed iron was present. The gall bladder was filled with black sludgy bile which contained one pigment stone (2 cm in diameter). It is usual to find pigment stones in the gall bladder in almost any kind of severe haemolytic anaemia, because an excess of bilirubin is formed and that means to say a great deal more bile pigment has to be got rid of than is normally present. The kidneys were not enlarged, they were very soft and the cortex was a rich brown colour (Plate XIX), and showed a stronger Prussian blue reaction than either the liver or the spleen. The medulla, by contrast, was a light crimson colour and there was only a very slight iron reaction in the medulla. There was early pyelonephritis with abscess formation in the cortex and medulla of both kidneys and that I think explains the urinary symptoms about which Dr. Crow was telling us. I grew some coliforms from the urine this time. I gather that on a previous occasion her urinary infection was *Proteus*. The renal pelves were natural. In the genital system the only abnormality was the fact that the oviducts had been obliterated by tying. The endocrines were normal, the bones were normal. I have described the meninges to you already and coronal section of the brain showed slight brown staining of an old infarct (4 cm each way) in the right parieto-temporal region. Her hemiplegia was a left one, was it not?

*Dr. Crow:* Yes.

*Dr. Lloyd:* It was an irregular infarct consisting of a central spongy mass destroying the right lenticular nucleus and extending through the internal capsule to the lateral wall of the left ventricle. It had therefore destroyed part of the conduction mechanism which was controlling the movement of the limbs on the left side of the body. She had a certain amount of recovery from her symptoms, but not complete.

(Dr. Lloyd then showed some coloured lantern slides of the organs and of microscopical preparations.) The renal cortex was much browner than the liver or spleen, because of its higher iron content. Prussian blue stains of sections of liver, spleen, kidney (Plate XX) and the bone marrow showed abundant iron, greatest in the tubular epithelial cells of the renal cortex. The glomeruli had escaped. The excreted haemoglobin was reabsorbed by the epithelium of the convoluted tubules and was there broken down, so that haemosiderin granules accumulated. The medullary tubules contained no iron. Plate XIX also shows streaks of medullary necrosis due to acute pyelonephritis. The cut surface of the femur, with dark brown-red marrow from end to end, resembled that seen in pernicious anaemia, and for the same reason. The brown component is haemosiderin from red-cell breakdown, the red is reactive hyperplasia of the marrow, mainly in this case of red-cell precursors. Myelocytes were not nearly as numerous as they ought to have been, probably because the main call on the bone marrow was to make red corpuscles. This explains the fall in her peripheral white cell count. The haemosiderin was in the macrophages. These cells were very active (Plate XXI), many of them were seen to be stuffed with red blood corpuscles. They have evidently acquired an avidity for red cells, which they have ingested and which they are in the process of destroying. That is another way in which blood was being destroyed in this particular patient.

The morbid anatomy of this condition was described by Bodley Scott and Robb-Smith in 1938. They described two cases and reviewed the literature of twelve other cases, a total of fourteen, beginning with a case of Marchiafava's in 1911. They described two particular things that are characteristic of this disease. One of those is present in this case. This case also has another characteristic which is not normally present in paroxysmal nocturnal haemoglobinuria and we shall see why that is so. Firstly the condition that is normally found in nocturnal haemoglobinuria, that is *not* present here, is the tendency to get micro-emboli composed of the red cell envelopes of haemolysed red cells. These have a tendency to block the terminal arteries and arterioles and to produce focal necrosis in a variety of organs, rather more conspicuously it seems in the liver. I was not able to find anything of that sort in this case. The thing which this case *does* show which is normally found in this disease is an extraordinarily large quantity of iron in the cortex of the kidney and the reason for that I have already explained to you. Normally you do not get any increase of iron either in the liver or in the spleen in paroxysmal nocturnal haemoglobinuria because the red cell break-down takes place in the kidney and not in the reticulo-endothelial system of the body. That has not been so in this case and that brings me to the thing in respect of which this case differs from all of those cases described in 1938, which you will appreciate is before the era of blood transfusion. In our case there is evidence that the reticulo-endothelial system, particularly the macrophages which I have shown you in the bone marrow, has a considerable avidity for red blood corpuscles. Whether they are the patient's own red blood corpuscles or whether they are transfused ones I do not know. But it is quite obvious that a good deal of red blood destruction is taking place other than in the kidney. The consequence of this is that the products of that destruction have been taken up by the reticulo-endothelial system particularly in the liver, spleen and bone marrow, and after a certain amount of redistribution in the liver they have come to lie within the parenchyma cells of the liver as well. It is the kind of change you commonly find in people who have had large numbers of transfusions for one reason or another. It may be they have had an aplastic anaemia which has necessitated large numbers of transfusions and therefore it is sometimes known as "transfusional siderosis". I have some figures which show the difference in the proportion of iron in these different conditions and in these different organs:—

### IRON ANALYSIS

BY MR. A. H. TINGEY

| | *Normal control* | *P.M.* 7478 | *Marchiafava's case* (1911) |
|---|---|---|---|
| *Liver* | | | |
| Water content percentage .. | 73.7 | 79.6 | |
| Available iron (mg. percentage wet basis) .. .. .. | 16.2 | 154 | 6.17 |
| *Spleen* | | | |
| Water content percentage .. | 78.2 | 81.5 | |
| Available iron (mg. percentage wet basis) .. .. .. | 6.5 | 285 | 9.26 |
| *Kidney* | | | |
| Water content percentage .. | 80.9 | 83.5 | |
| Available iron (mg. percentage wet basis) .. .. .. | 17.2 | 415 | 86.9 |

("Available iron" means all iron except that due to blood).

You will see that in Marchiafava's case the proportion of iron in the liver and in the spleen is pretty close to normal but that there is a considerable excess of it in the kidney and only in the kidney. That corresponds with the findings of all the other cases where they found people getting a strong Prussian blue reaction in the renal cortex but not in any of these other organs. In our particular case here though it is not so. You can see a considerable excess of iron both in the liver and in the spleen although there is twice as much in the kidney as there is in either of the other two organs. Those are the principal findings.

*Professor Hewer:* I think this might be a good place to take the anomalies. Would Dr. Tovey like to say whether he thinks the large amount of iron in the liver and spleen here is the result of transfusion, and whether that complicates the issue?

*Dr. Tovey:* Yes, I think Dr. Lloyd's suggestion is a very happy one. I had not thought about this problem before and I am sure he must be right. Regarding the macrophages engulfing cells—these are presumably the transfused cells and this might be the first evidence that the patient was developing antibodies to the transfused cells. It is under such circumstances that red cells are engulfed by the macrophages.

*Professor Hewer:* Would Dr. Raper tell us what he now thinks is the explanation of the nocturnal character of the disease? Do you feel that these American experiments which you mentioned are against any possibility of increased $CO_2$ at night?

*Dr. Raper:* I think that as a *single* explanation it is probably ruled out by these experiments. Other things happen in sleep no doubt . . .

*Dr. Sanerkin:* What happens to these patients if they sleep during the daytime and work at night?

*Dr. Raper:* It does eventually follow the sleep and not the sun.

*Question:* Was there any iron in the pancreas?

*Dr. Lloyd:* No, there was none.

*Question:* How much of this iron present in the liver and spleen could be explained in terms of the iron which this patient received?

*Dr. Raper:* I do not know quite how much she had, but a person with haemolytic anaemia who is treated with iron would certainly lay it down.

*Dr. Crow:* I have not worked out how much she had, she certainly was given very large amounts of iron by mouth, and had several courses of intramuscular iron, but I

did not take particular note of how much the total was. We have not got any Prussian blue reaction at the site of imferon injection?

*Dr. Lloyd:* No, I am afraid that those particular sites were not identified by me. Was she actually having those injections just before coming to me?

*Dr. Crow:* Oh no, it was a good many years ago, at least 5 or 6, probably more, that she had imferon.

*Dr. Raper:* We have not to condemn it in this condition (although of course we could in other haemolytic conditions) because here there is a loss of iron. It is remarkable that she should have had so much iron in her organs and yet apparently in the phases between her haemolytic bouts her anaemia appeared to be iron deficient. Is this iron which is deposited in these tissues not utilised?

*Dr. Crow:* I think that iron laid down as a result of transfusion is not readily available. Haemosiderin is apparently resistant to break-down and re-utilisation.

*Dr. Tovey:* One of the further characteristics of this disease not yet mentioned is that not all the red cells are abnormal and that this seems to vary with the individual patient and with the time when we see the patient. When for example, the patient is having a relatively good phase only about 10 per cent of the cells may appear abnormal under electron microscopy but in a bad phase up to 90 per cent. Dacie has queried whether this means that such patients develop abnormal clones which then produce abnormal red cells, so that there are phases in which the patient produces relatively normal red cells and other phases in which the abnormal clones take over.

*Professor Hewer:* Will you tell us what is the electron microscopic abnormality of those that are abnormal?

*Dr. Tovey:* I have not seen these myself, I think Dr. Raper has.

*Dr. Raper:* I have not seen it, I have seen reports only. The surface is partially pitted; it looks almost like the skull in marked myeloma.

*Question:* Is this disease inherited or acquired?

*Dr. Tovey:* It is certainly not inherited, but in some cases the first attacks are apparent as early as the second decade. I remember one patient who started his first attack at the age of 24.

*Question:* What is the usual age for presenting? Is this the normal pattern of the disease? Does it present at an early age?

*Dr. Tovey:* I would not like to be dogmatic about that.

*Dr. Crow:* I can tell you what the textbook says, "it occurs at any age, it begins at any age except childhood, but is commonest in the third decade". The unusual thing with this patient is the time which she lasted. Again the textbook will tell you that the prognosis is not very good and the patient will succumb within a few years. This patient lived from the age of 21 to 57 with the disease.

*Professor Hewer:* Thanks to transfusions do you think?

*Dr. Crow:* Well partly thanks to that. You may have noticed that when I spoke of treatment I did not refer to any treatment that had any influence on her haemolysis. We were entirely in the hands of the disease. We could keep pace to a certain extent by transfusing her but nothing we could do appeared to influence the haemolysis. Fortunately she always did settle down shortly after coming into hospital. She was an odd woman; she was apt to hide herself away when she had a bad spell so that she usually did not appear at the hospital until her haemoglobin was down to 50 per cent or below. That was possibly just as well for us, because it meant that we usually got her at the end of a haemolytic phase. It may be that actually coming into hospital and resting in bed had a beneficial effect on her haemolytic crisis.

*Dr. Raper:* Yes, when you rest such people their red cells are subjected to less buffeting are they not? The other thing that does help in cases of this type is that when the patient is transfused bone marrow activity is depressed, so that you get fewer of their abnormal cells being produced for a while. We found that very striking in this and in another case. He used to go into hospital fairly regularly with advanced haemoglobinuria and Hb down to about 40 per cent, and after transfusion his haemoglobinuria would cease very dramatically. We could not explain this for a long time. Now it is quite clear what was happening; with the bone marrow depressed by the transfusion he was not producing the abnormal cells.

*Dr. Crow:* (in answer to a question): Apparently there is a very delicate balance between the haemolysins and the inhibitors. There are said to be two of each, (one of the haemolysins is thrombin itself) and the picture I get from reading of it is that there is a very delicate balance, and it does not take much to upset it. Although the defect is in the red cell itself, all these things somehow apparently are implicated. It used to be thought that it was the pH purely but obviously that is not so. There must be much more to it than that.

There is one other aspect of this that I was hoping to hear about and since it may have some therapeutic application it ought to be aired tonight. I think it was Israel and Wilkinson from Manchester who some years ago suggested in the *Lancet* that infusions of Dextran could bring to an end a haemolytic crisis. Dr. Raper, I think you will have to help me, I think it is something to with properdin?

*Dr. Raper:* This may come into the question that was asked about infections. As you know, one of the substances that deals with bacteria in the blood stream in a quite nonspecific way is a substance known as properdin, the thing that "prepares them for destruction". It is not an immune antibody, and it has been shown that this stuff, strangely enough, is one of the things that aids in the lysis of P.N.H. cells. In fact the action of thrombin, which I was mentioning, has been shown to be due merely to properdin adsorbed upon it. Properdin certainly varies in amount in different people, and there has even been a case of paroxysmal nocturnal haemoglobinuria who lacked the normal amount of properdin and was consequently very mildly affected; apart from that I cannot tell you very much about it. I do not know how this can be developed therapeutically and I do not know what the effect of Dextran would be.

*Dr. Crow:* This was only a preliminary communication that I read recently and although I think it was in 1958 I can find no trace of any subsequent publication. But they had found that Dextran in certain concentrations reduced the amount of properdin in the serum of normal patients, and they utilised this means of reducing the properdin in the serum of patients with paroxysmal nocturnal haemoglobinuria by giving them intravenous Dextran. They did at that time try it on two patients and it appeared to arrest the haemolysis but as has already become clear tonight you take the patient into hospital and put him to bed and this appears to arrest the haemolysis anyway. From the fact that from 1958 to 1962 no subsequent publication has appeared I would think that it probably does not work.

*Dr. Tovey:* One of the hazards of Dextran is that it may add to the kidney damage and I think that is one of the reasons why it has not been further pursued in this condition.

*Professor Hewer:* One of the things that happens in all sorts of infections is that the steroid disappears from the cortex of the adrenal. I think you mentioned steroids. Is there any possibility that the adrenal cortex might be affected by anxiety as well as by infections?

*Dr. Raper:* Wayne of Glasgow, the pharmacologist, tried ACTH and cortisone separately in these people and found that it didn't work.

*Question:* Has anybody tried keeping these people away from the situations which precipitate haemolysis? I understand she had an unhappy married life? If she could be taken away from this, might not the episodes be prevented?

*Professor Hewer:* She was put to bed in the Infirmary and did admirably there.

*Dr. Crow:* The average human being, however unfortunate his situation, would usually rather be in his own situation than somewhere else.

*Question:* How often does this disease occur? Is it not very rare?

*Dr. Raper:* It is rare certainly.

*Dr. Crow:* It always goes on the end of the list when discussing haemolytic anaemias, anyway.

*Dr. Tovey:* We have treated four cases in Bristol in the last decade so that although it is rare, it is not all that rare.

*Professor Hewer:* I rather think that concludes the discussion. It seems to me it was interesting not only because it was a rare form of haemolytic anaemia but it brought up lots of factors common to all haemolytic diseases.

## Figures and Tables

**Figure f1:**
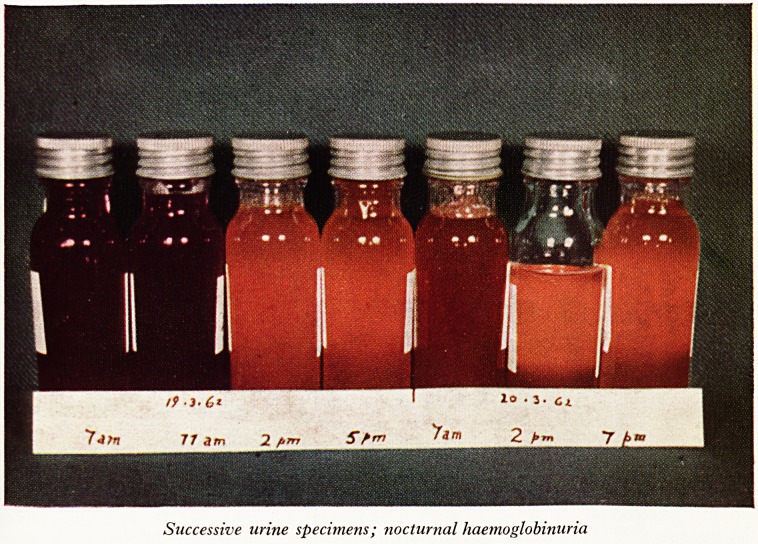


**Figure f2:**
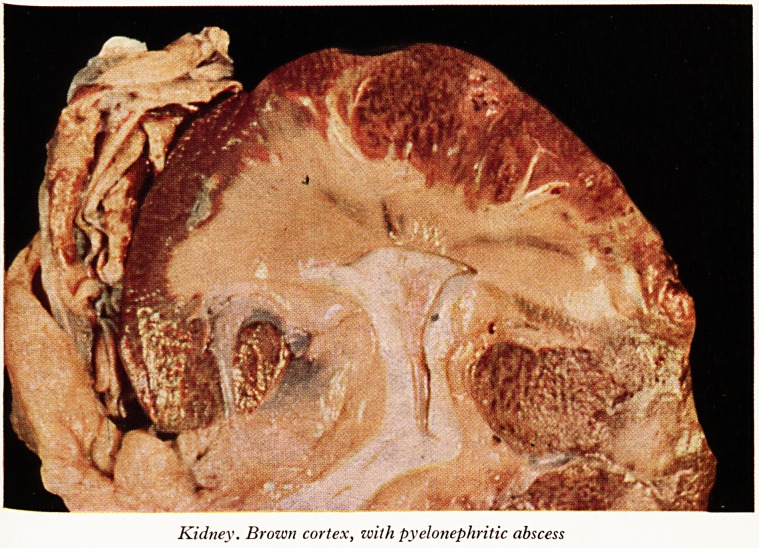


**Figure f3:**
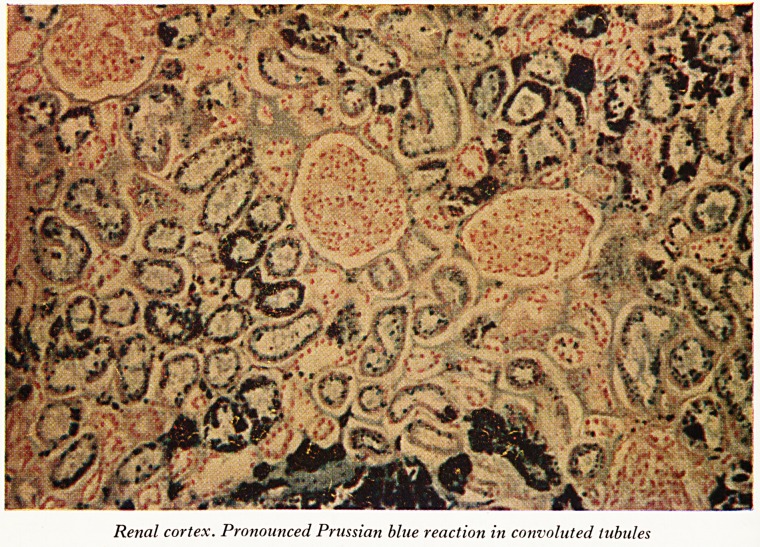


**Figure f4:**